# Human Bone Marrow Mesenchymal Stem Cell Behaviors on PCL/Gelatin Nanofibrous Scaffolds Modified with A Collagen IV-Derived RGD-Containing Peptide

**Published:** 2014-02-03

**Authors:** Ali Mota, Abbas Sahebghadam Lotfi, Jalal Barzin, Mostafa Hatam, Behzad Adibi, Zahra Khalaj, Mohammad Massumi

**Affiliations:** 1 Department of Clinical Biochemistry, Faculty of Medical Sciences, Tarbiat Modares University, Tehran, Iran; 2 National Institute of Genetic Engineering and Biotechnology (NIGEB), Tehran, Iran; 3 Iran Polymer and Petrochemical Institute (IPPI), Tehran, Iran

**Keywords:** Nanofibers, Polycaprolactone, RGD Peptide, Surface Modification, Mesen- chymal Stem Cell

## Abstract

**Objective:**

We introduce an RGD (Arg-Gly-Asp)-containing peptide of collagen IV origin that possesses potent cell adhesion and proliferation properties.

**Materials and Methods:**

In this experimental study, the peptide was immobilized on an electrospun nanofibrous polycaprolactone/gelatin (PCL/Gel) hybrid scaffold by a chemical bonding approach to improve cell adhesion properties of the scaffold. An io- dine-modified phenylalanine was introduced in the peptide to track the immobilization process. Native and modified scaffolds were characterized with scanning electron microscopy (SEM) and fourier transform infrared spectroscopy (FTIR). We studied the osteogenic and adipogenic differentiation potential of human bone marrow-de- rived mesenchymal stem cells (hBMSCs). In addition, cell adhesion and prolifera- tion behaviors of hBMSCs on native and peptide modified scaffolds were evaluated by 3-(4,5-dimethylthiazol-2-yl)-2,5-diphenyltetrazolium bromide (MTT) assay and 4',6-diamidino-2-phenylindole (DAPI) staining, and the results compared with tissue culture plate, as the control.

**Results:**

FTIR results showed that the peptide successfully immobilized on the scaffold. MTT assay and DAPI staining results indicated that peptide immobilization had a dramatic effect on cell adhesion and proliferation.

**Conclusion:**

This peptide modified nanofibrous scaffold can be a promising biomaterial for tissue engineering and regenerative medicine with the use of hBMSCs.

## Introduction

In recent years a wide range of polymeric biomaterials with various properties have been developed for bioengineering and tissue engineering applications. These biomaterials should be biodegradable, bioactive and biocompatible (1). The biomaterials architecture is also important and affects the cellmatrix interaction (2), which is of significance in cellular behaviors such as cell adhesion, proliferation, and differentiation (3). Cell adhesion is the first event in the cellular response to biomaterials (4). Many of these biomaterials exhibit desired biodegradation characteristics and reasonable mechanical properties (5). One of the challenging issues regarding polymeric materials such as polycaprolactone in biomedical applications is that they do not possess necessary bioactive and biomimetic characteristics to interact with seeded cells (6). Approaches to improve the biomimetic properties of such biomaterials include materials modification by hybridization with bioactive compounds and/or immobilization of bioactive motives to enhance and control interaction between cells and synthetic biomaterials (7).

Electrospinning is a simple and highly throughput technique for producing hybrid nanofibers with suitable high porosity and surface area structure to mimic nanoscaled patterns of natural extracellular matrix (ECM) to control cell behavior (2). In recent years several attempts have resulted in the production of polymeric nanofibrous scaffolds using electrospinning for biomedical applications (8, 9). Hybridization of biomaterials with bioactive substances such as gelatin (10, 11) collagen (12, 13), and fibroin (14) have been used in numerous studies to modulate the biomimetic properties of polymeric materials for a variety of applications. Immobilization of cell recognition peptides on biomaterials (15, 16) or peptide amphiphils (17, 18) accelerates improvement of the biomaterial’s surface chemistry. Cell-cell and cell-ECM interactions are mediated by cell adhesion receptors. The integrin family is among the most versatile group of cell adhesion receptors. They are involved not only in cell anchoring but also in numerous other processes such as proliferation, differentiation, and homeostasis (19). The RGD (Arg-Gly-Asp) sequences are the most prominent cell recognition sequences present in many ECM proteins (20). It has been demonstrated that approximately half of the integrin family binds to ECM proteins through the RGD sequences. Therefore, the RGD peptides are by far the most effective peptides for improving cell adhesion to biomaterials surfaces.

We introduced and synthesized a novel RGDcontaining peptide of collagen IV origin and immobilized the peptide on the surface of a hybrid polycaprolactone/gelatin (PCL/Gel) nanofibrous scaffold. The morphology and structure of the PCL/ Gel nanofibrous scaffold were evaluated by scanning electron microscopy (SEM). We characterized the RGD-modified scaffold with fourier transform infrared spectroscopy (FTIR). Behaviors of human bone marrow-derived mesenchymal stem cells (hBMSCs) including cell adhesion and proliferation on the RGD-modified PCL/gel nanofibrous scaffold were studied by 3-(4,5-dimethylthiazol-2-yl)-2,5- diphenyltetrazolium bromide (MTT) assay and compared with non-modified scaffold and tissue culture plate (TCP) as the control. Cell adhesion to scaffolds was visualized by 4',6-diamidino-2-phenylindole (DAPI) staining of the cell nucleus.

## Materials and Methods

In this experimental study, Fmoc (9-fluorenylmethoxycarbonyl)- protected amino acids with various protected side chains, rink-amide-MBHA resin, and O-(benzotriazole-1-yl)-N, N, N´, N´-tetramethyluronium-hexafluorophosphate (HBTU) were purchased from GL Biochem (Shanghai, China). HPLC grade trifluoroacetic acid (TFA), acetonitrile (ACN) and water, N-ethyldiisopropylamine (DIEA) and triisopropylsilane (TIPS) were products of Merck Chemicals (Germany). Gelatin type B powder (300 g Bloom) from porcine skin was prepared from Sigma (USA). PCL (Mn 80,000) and 1-ethyl-3-(3-dimethylaminopropyl) carbodiimide (EDAC) were obtained from Sigma-Aldrich (St. Louis, MO, USA). Dulbecco Modified Eagle’s Medium (DMEM), fetal bovine serum (FBS), phosphate buffered saline (PBS) and trypsin/EDTA were products of Gibco (Invitrogen, USA). 3-(4,5-dimethylthiazol-2-yl)-2,5- diphenyltetrazolium bromide (MTT) and 4',6-diamidino- 2-phenylindole (DAPI) were purchased from Promega (USA). All other chemicals and solvents were obtained from Merck unless otherwise stated and used without further modifications.

### Peptide synthesis

A 10-mer RGD-containing peptide of collagen IV origin with a sequence of KK-[GPRGDPG]- F(4-I) and a theoretical mass of m/z 1183.1 was synthesized by standard Fmoc chemistry using an Advanced Chemtech 90 peptide synthesizer (USA). The peptide was synthesized on rinkamide- MBHA resin with a theoretical loading of ≈0.55 mmol/g. The total loading of the resin was used for all calculations. Standard Fmoc-protected amino acids (4 equivalents) were used in each coupling reaction. Coupling was performed for 15 minutes with a mixture of DIEA (2.5 equivalents) and HBTU (0.98 equivalent). A solution of 20% (v/v) piperidin in DMF was used for the deprotection reaction. Coupling and deprotection reactions were confirmed by the Kaiser test. After final deprotection, the resin was washed 3 times with methanol and dried in a vacuum chamber. A cleavage cocktail mixture of 95% TFA, 2.5% TIPS and 2.5% deionized water for 2 hours on ice was used for cleavage of the peptide from the resin. Cleaved peptide was precipitated by cold diethylether and resolubileized in deionized water. A modified iodinated phenylalanine (4-I-phenylalanine) at the carboxyl end of the peptide was used in order to confirm the immobilization of the peptide on the surface of the PCL/Gel nanofibrous scaffold.

### Peptide purification and characterization

The purity of crude peptide was analyzed by reverse-phase high performance liquid chromatography (RP-HPLC; Agilent, USA). RP-HPLC was done on an octadecylsilica (C18) column (4.6 mm id×250 mm length, 5 μm) with TFA/water (0.1% v/v) and TFA/ACN (0.1% v/v) as the mobile phase with a linear gradient from 0 to 60% of TFA/ACN over 30 minutes and flow rate of 1 mL/minute at 25˚C. RP-HPLC results were used to scale up and purify the peptide. The peptide was purified by preparative HPLC (Agilent, USA) with >95% purity with the same mobile phase as RP-HPLC. Purified peptide was lyophilized and stored at -20˚C until future use in the experiments. For characterization of the synthesized peptide, mass spectrum of the peptide was obtained by quadropole LC-MS (Agilent, USA) with an electrospray ionization system.

### Electrospinning

2, 2, 2-trifluoroethanol (TFE) was used as a common solvent for PCL and gelatin. PCL granules and gelatin powder were dissolved separately in TFE by stirring overnight at room temperature to prepare 10% w/v solutions. A PCL/Gel blended solution at a ratio of 1:1 was loaded into a 10 mL syringe. The syringe was attached to the pump and the blended solution delivered by a steady flow of 1 mL/hour to a 22G stainless steel blunt needle by a plastic tube. A high voltage of 22 kV was applied to the needle and the solution was electrospun for 4 hours. The electrospun hybrid nanofibers were collected on a rotating drum collector on aluminum foil that was placed at a distance of 15 cm from the tip of the needle. The collector rotation speed was set at 100 rpm and shuttling speed at 5 mm/ minute. The electrospinning process was done at 25˚C and 50% humid atmosphere. The electrospun nanofibers were dried in a vacuum overnight and named PCL/Gel.

### Scaffold cross-linking

Electrospun PCL/Gel nanofibrous scaffolds were cross-linked using a solution of EDC/NHS in ethanol/ water (80:20 v/v) as previously described (21). The cross-linking process was done by placing the air-dried PCL/Gel hybrid scaffolds together with a supporting aluminum foil in a 250 mM solution of EDC/NHS for different time periods. The extent of cross-linking was assessed for the presence of free amine group content by the ninhydrin assay. A sample of each scaffold was incubated with 0.3 M ninhydrin solution in ethanol for 5 minutes at 95˚C. The first sample that become negative according to the ninhydrin test after a certain period of time was considered to be a complete crosslinking process. This complete cross-linked scaffold was used in the next step.

### Peptide immobilization

Cross-linked scaffolds were rinsed 3 times with PBS to remove excess EDC/NHS and the scaffolds were immersed immediately 1 μmol/mL of the peptide in PBS and incubated for 24 hours with mild stirring at 25˚C (22). Functionalized scaffolds were then washed 3 times with PBS and dried under vacuum for 24 hours.

### Fourier transforms infrared spectroscopy (FTIR)

For surface characterization and chemical analysis of scaffolds and confirmation of the immobilization of the peptide on nanofibers, FTIR spectroscopy of PCL/Gel and RGD-modified PCL/Gel scaffolds were performed over a range of 4000 to 400 cm^-1^ at a resolution of 4 cm^-1^ using a Bruker (Bruker, Tensor 27, USA) FTIR system. To assign a certain peak to the iodine tag, FTIR spectra of phenylalanine and 4-iodophenylalanine as controls were also recorded.

### Scanning electron microscopy (SEM) analysis

The morphology and structure of electrospun PCL/ Gel and RGD-modified PCL/Gel nanofibers were analyzed by SEM. Air-dried samples were sputter coated with gold in a KYKY sputter coater (KYKYSBC- 12, China) prior to analysis. SEM images were recorded with KYKY-EM-3200 SEM system (China) at an accelerating voltage of 25 kV. The distribution of electrospun nanofibers diameter was analyzed by measuring at least 100 fibers using Image J software (National Institutes of Health, USA)

### Human bone marrow-derived mesenchymal stem cells (hBMSCs) culture

hBMSCs were isolated from the iliac crests of healthy donors at Royan Institute (Royan Institute Cell Bank). All samples were collected followingdonors’ informed consents. The study was approved by the Ethical Committee of Tarbiat Modares University. hBMSCs were grown on DMEM medium supplemented with 10% FBS and 1% penicillin (10000 U/mL)/streptomycin (10 mg/mL). Cells were subcultured to 80% confluency and incubated at 37˚C in a humidified atmosphere of 5% CO2. Cells were used for osteogenic and adipogenic differentiation and cell adhesion and proliferation studies at passage 4.

### Osteogenic differentiation

To induce osteogenic differentiation, upon reaching 80% confluency, hBMSCs were detached using 0.05% trypsin/EDTA and plated in 4-well culture dishes at a density of 5000 cells/well and were treated with osteogenic medium (DMEM supplemented with 10% FBS, 0.1 mmol/L dexamethasone, 10 mmol/L glycerol phosphate, and 0.2 mmol/L ascorbic acid) for 14 days. The medium was changed twice weekly. The cells were fixed with 4% paraformaldehyde for 10 minutes and washed with phosphate buffered saline (PBS). Osteogenesis was assessed by alizarin-red staining for visualizing deposition of calcium (9).

### Adipogenic differentiation

For adipogenic differentiation, hBMSCs were seeded in 4-well culture plates at a density of 5000 cells/well. The cells were treated with adipogenic medium (DMEM supplemented with 10% FBS, 0.5 mmol/L 3-isobutyl-1-methylxanthine, 1 μmol/L dexamethasone, 1.7 μmol/L insulin and 200 μmol/L indomethacin), for 2 weeks. Medium was replaced twice weekly. The cells were fixed with 4% paraformaldehyde for 10 minutes and washed with 70% ethanol. Adipogenesis was assessed by oil red O staining for visualizing deposition of fat droplets in differentiated cells (9).

### Cell adhesion and proliferation studies

The interaction between cells and scaffolds on PCL/Gel, RGD-modified PCL/Gel and TCP as control were studied using a colorimetric MTT assay. Cross-linked and RGD-immobilized PCL/Gel scaffolds were cut with a paper hole punch, transferred into 96-well plates (Nunc, Denmark), and then sterilized with 70% ethanol for 30 minutes. Ethanol was aspirated and the scaffolds were dried and further sterilized for 15 minutes under UV prior to cell seeding. hBMSCs were trypsinized and plated at 5000 cells/well. The cells were incubated for 1, 2, 3 and 4 hours for cell adhesion and 72 hours for the cell proliferation and viability assay at 37˚C in a 5% CO2 and 95% humidified atmosphere in DMEM supplemented with 10% FBS. At each time point, the medium was discarded and the scaffolds were washed 3 times with PBS to remove non-adhered cells. The adhered cells were then incubated with MTT solution (10% (v/v) MTT stock (1 mg/mL) in DMEM) at 37˚C for 2 hours. Afterwards, the attached cells were washed twice with PBS. The developed purple formazan precipitates were solubilized and extracted with DMSO. The absorbance of each well was measured at 570 nm using a plate reader (Tecan, Switzerland). hBMSCs cultured on a blank plate were used as the control. The absorbance of solubilized formazane crystals is directly attributed to the number of adhered live cells. To verify the presence of cell adhesion on scaffolds, DAPI staining was done. In brief, in another set the scaffolds were washed with PBS 3 times and the attached cells were fixed with 4% paraformaldehyde for 10 minutes. The scaffolds were washed 3 times with PBS for 5 minutes. DAPI (1:1000 dilution) was added onto the scaffolds and incubated for 5 minutes. Fluorescent images were recorded by a Nikon TE2000 microscope.

### Statistical analysis

Statistical analysis was performed with SPSS, version 17 (SPSS Inc., USA). All cell culture experiments were performed in triplicate. Mean ± standard deviations were used for adhesion and proliferation data analyses. To compare mean values in different scaffolds analysis of variance (ANOVA) was used. Comparisons were performed by Tukey’s post HOC test. A p value of <0.05 with 95% confidence interval was considered as statistically significant.

## Results

### Electrospinning

**Fig 1:**
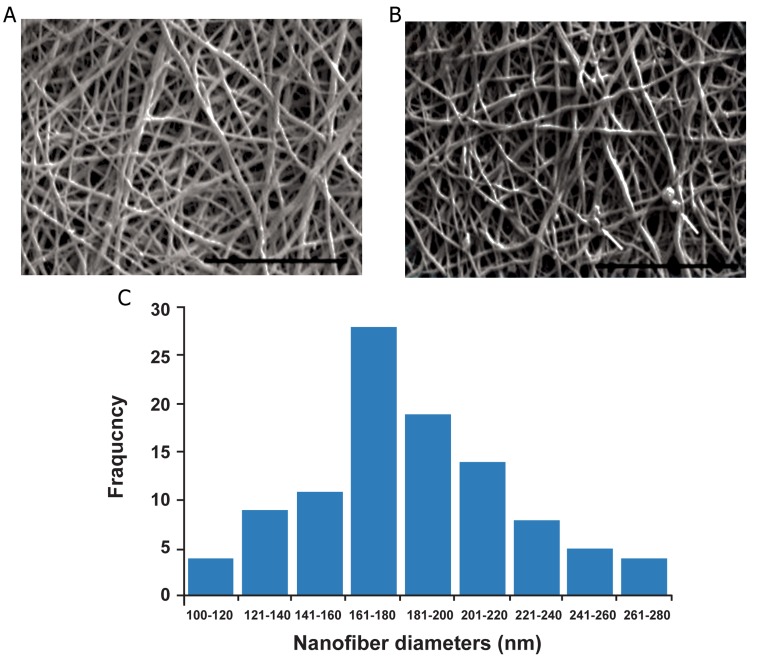
Scanning electron microscopy (SEM) images of nanofibrous scaffolds electrospun from 10% (w/v) PCL/Gel blended solution (A) before and (B) after cross-linking. Arrow shows some artifacts due to precipitation of phosphate buffered saline (PBS). Distribution of fiber diameter in electrospun PCL/Gel scaffolds (C). Scale bar: 10 μm. Resolution: ×7500.

We successfully fabricated PCL/Gel scaffolds of the desired porosity and nanofiber properties. SEM micrographs confirmed the homogenous morphology of the PCL/Gel nanofibers (Fig 1A). The SEM images in figure 1B show no dramatic changes in the morphology and diameter of the nanofibers after cross-linking and peptide immobilization. These nanofibers had a randomly oriented structure where the majority of mats had nano-scale diameters between 160 to 200 nm (190 ± 38 nm, Fig 1C).

### Peptide characterization

**Fig 2:**
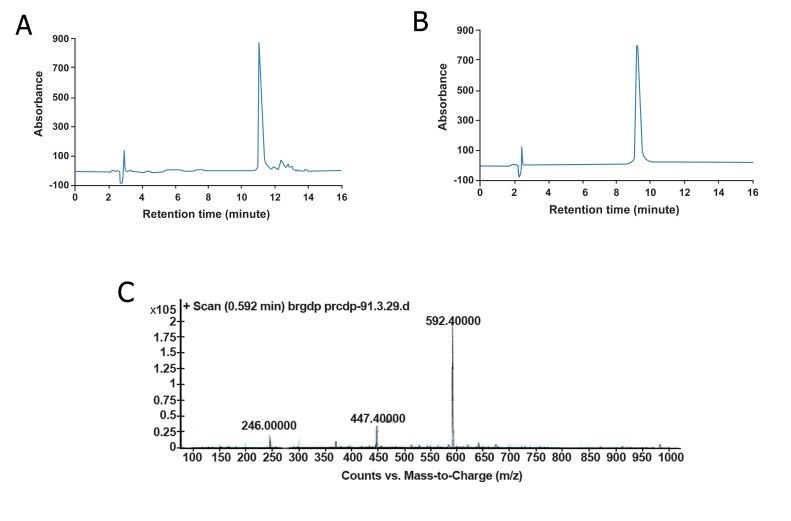
A. Analytical and B. preparative HPLC chromatogram for analyzing and purification of the synthesized peptide. C. Mass spectrum for analyzing peptide mass.

We successfully synthesized an RGD-containing peptide with cell attachment properties and an iodine tag to track peptide immobilization. As shown in figure 2A, the crude peptide HPLC chromatogram showed some impurities which were omitted after purification with preparative HPLC (Fig 2B). Mass spectrometry analysis showed the main peak with the corresponding m/z ratio of 592.4 which closely approximated the theoretical mass of the peptide (1183.1), assuming the peptide obtain two positive charge in the process of sample preparation (Fig 2C).

### Scaffold cross-linking

**Fig 3:**
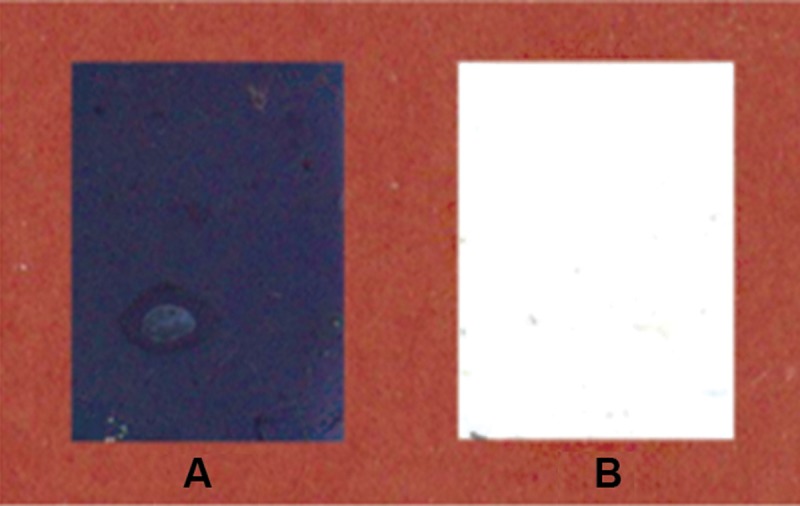
Ninhydrin staining of PCL/Gel scaffold. A. On the native scaffold, ninhydrin produced a dark blue color when reacting with free amine groups present in gelatin lysine residues. B. After cross-linking these amine groups coupled to free carboxyl groups and the scaffolds became colorless.

In the cross-linking process, the carboxyl groups present on gelatin were activated when the scaffold was treated with EDC/NHS and amide bonds formed between the carboxyl and amine groups in gelatin. The ninhydrin test is an indicator of the presence of free amine groups. As shown in figure 3 the scaffolds were stained dark blue before cross-linking. After complete cross-linking, the ninhydrin test was negative (Fig 3).

### RGD immobilization and scaffold characterization

**Fig 4:**
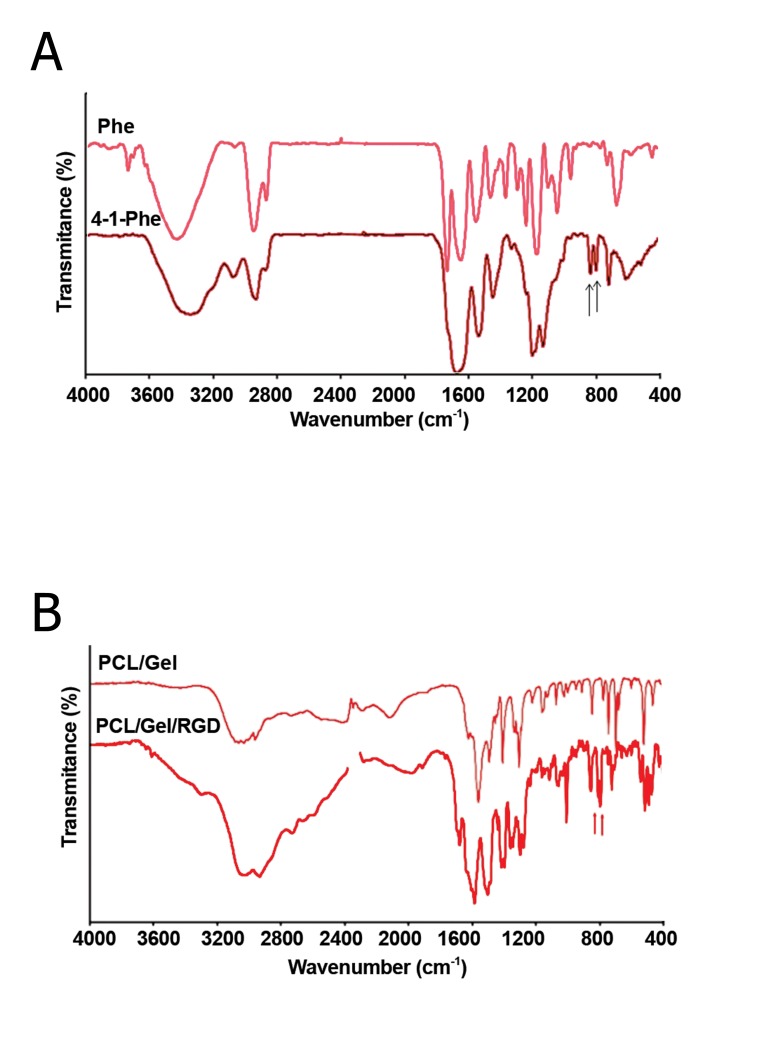
A. Fourier transform infrared spectroscopy (FTIR) of Phe and 4-Iodo-Phe standards. Arrows show the characteristic peaks assigned to C-I bond stretching. B. FTIR spectroscopy of PCL/Gel and RGD modified PCL/Gel. The assigned peaks are close to 800 cm^-1^ on the RGD modified PCL/Gel scaffold spectrum.

The FTIR spectrum of phenylalanine and 4-Iphenylalanine are shown in figure 4A. FTIR spectrum of 4-I-phenylalanine showed characteristic peaks at 798 cm^-1^ and 812 cm^-1^ that could be ascribed to the C-I bond since the C-I bond led to the appearance of some peaks close to 800 cm^-1^. These significant peaks were not present in the FTIR spectrum of the PCL/Gel (Fig 4B). The characteristic peaks attributed to the C=0 and N-H bond were present at 3500 cm^-1^ (C=O) and 3300 cm^-1^ (N-H). The peak at 1650 cm^-1^ was assigned to the amide I that corresponded to gelatin in the PCL/Gel (23). The FTIR for PCL/Gel scaffold modified with RGD peptide revealed a characteristic peak at 798 cm^-1^. The other peak at 812 cm^-1^ was also present with some delocalization to 840 cm^-1^ (Fig 4B). The presence of these peaks implied that the RGD peptide immobilized on the PCL/Gel scaffold.

### Osteogenic and adipogenic differentiation potential

Figure 5A shows the characteristic lipid droplets stained by oil-red-O staining. According to the results of alizarin red staining (Fig 5B), hBMSCs cultured in osteogenic differentiation medium deposited a mineralized matrix after 14 days of incubation. The results of this experiment showed that hBMSCs could differentiate into osteocytes and adipocytes.

**Fig:**
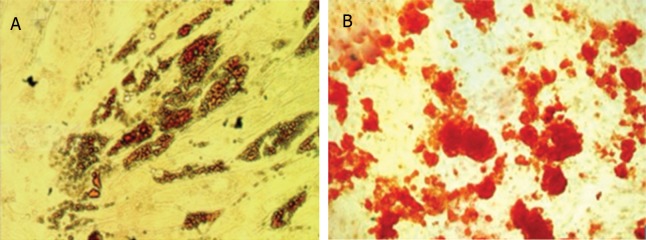
Adipogenic and osteogenic differentiation potential of hBMSCs. A. Lipid droplets are present in differentiated hBMSCs and stained red by oil-red-O staining. B. Calcium crystals are shown in differentiated hBMSCs by alizarin red staining.

### Cell adhesion and proliferation studies

**Fig 6:**
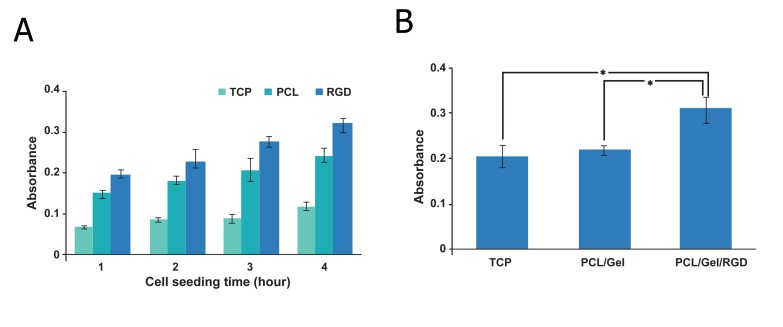
A. MTT assay results of hBMSC adhesion on different scaffolds at 1, 2, 3 and 4 hours after cell seeding. There are significant differences in cell adhesion between scaffolds at each time point. B. MTT assay for hBMSC proliferation on different scaffolds. After 72 hours of cell seeding RGD modified PCL/Gel scaffold demonstrated a dramatic effect on hBMSC proliferation according to the MTT assay (*; p≤0.5). No significant difference was found between PCL and TCP in their effects on cell proliferation (p>0.5).

Figure 6 shows the status of hBMSC adhesion on different scaffolds. Cell adhesion and proliferation were measured by the MTT assay. It was clearly obvious that cell adhesion on TCP did not seem to increase significantly at different time points (p>0.05). hBMSCs adhered rapidly on both PCL/Gel and RGD modified PCL/Gel scaffolds (Fig 6A). The adhesion increased gradually in a time-dependent manner. Immobilization of RGD peptides on PCL/Gel nanofibrous scaffolds resulted in increased adhesion behavior of the scaffold. There was more adhered cells on the PCL/Gel scaffold grafted by RGD compared to those cultured on TCP and non-grafted PCL/Gel (p≤0.05). Adhesion of cells on RGD-modified PCL/Gel also gradually increased at different time points. TCP did not show a time-dependent adhesion. We found that cell adhesion on TCP slightly increased over time.

**Fig 7:**
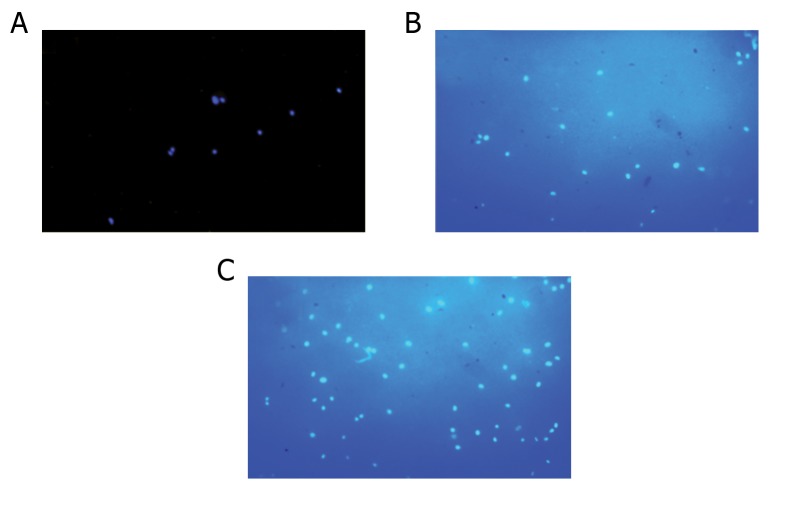
DAPI staining for visualization of attached cells on: A. tissue culture plate (TCP); B. native PCL/Gel scaffold; and C. RGD-modified PCL/Gel scaffold. Clearly, RGD-modified PCL/Gel scaffold showed a significantly greater number of bound hBMSCs after cell seeding compared with TCP and the unmodified scaffold.

While PCL/Gel did not show significant effects on hBMSCs proliferation (p>0.05), it was clearly apparent that immobilization of RGD had a very significant effect on cell proliferation (p≤0.05, Fig 6B). DAPI stained images showed that a greater number of hBMSCs attached on the RGD-modified scaffold compared with TCP and the unmodified scaffold (Fig 7).

## Discussion

We synthesized a collagen IV-derived RGDcontaining peptide that had cell adhesion and proliferation properties. The peptide was successfully immobilized on the surface of electrospun hybrid nanofibrous PCL/Gel scaffolds. The RGDcontaining peptide was immobilized on scaffolds through covalent bonds without further chemical treatment after cross-linking to enhance the adhesion of hBMSCs and improve biomimetic properties of the scaffold.

Morphology and surface chemistry of nanofibrous scaffolds used in tissue engineering and regenerative medicine have an important impact on cell behaviors such as adhesion, proliferation, differentiation and cell-matrix interaction (24). The most challenging issue regarding polymeric materials such as PCL are the inadequate and nonspecific interactions that occur between polymers and cells (25). Most synthetic polymers such as PCL are hydrophobic and lack functional active groups such as hydroxyl, carboxyl, amine and sulfate groups to interact with cells. Many surface modification strategies have thus been developed to introduce such functional active groups on the surface of PCL.

Since the PCL surface lacks active groups such as amine or carboxyl groups, it cannot be easily modified. Alkaline hydrolysis and aminolysis are suitable methods for introducing these functional groups. The use of these methods, however, alters the structural and morphological properties of PCL nanofibers and can lead to formation of unstable nanofibers in terms of mechanical and morphological properties (26). To produce reactive groups, PCL is usually blended or surface-activated with other biomolecules such as collagen, elastin, fibroin, and surface activating peptides. Gelatin, which is derived from collagen, is cheaper and can be used to overcome this incompetency.

In the present study, we physically blended PCL with gelatin. We found that the cross-linked hybrid PCL/Gel scaffold had improved cell adhesion properties. This resulted from the presence of highly polar and functional groups of gelatin on the surface of electrospun nanofibers. The effect of hybrid PCL/Gel on cell behavior has been studied in previous works. PCL/Gel nanofibrous scaffolds can enhance differentiation of cerebellar stem cells toward functional nerve cells (27). Hybrid scaffolds composed of PCL and collagen are constructed and used in vascular reconstruction. Tillman et al. (28) have shown that PCL/collagen electrospun scaffolds maintained high potential and integrity in vivo without an abnormal inflammatory response. They concluded that this hybrid electrospun scaffold might have clinical applications. McClure et al. (29) used a three layered electrospun matrix to mimic native arterial structure using three different materials, PCL, elastin and collagen. They predicted that this three layered vascular graft contained overall properties within the range of the native artery.

Electrospinning of PCL was successfully performed with polar solvents in order to facilitate the electrospinning process. Gelatin, as a denatured form of collagen, has been used in many types of biodegradable and biocompatible scaffolds. A disadvantage for gelatin itself, or in combination with other biomaterials is its swelling in aqueous medium. Gelatin may be cross-linked to solve this problem by chemical cross-linkers such as glutaraldehyde and formaldehyde. However, these cross-linkers have been shown to be toxic (30) and disrupt the electrospun morphology (26). EDC is an effective cross-linker which introduces crosslinks that do not release any toxic end product or a foreign bond in cross-linked gelatin. It has been shown that EDC as a water-soluble carbodiimide can be used in gelatin cross-linking. Once it is used in an ethanol-water mixture, the swelling of gelatin can be prevented even in the cross-linking process (21).

Cross-linking of scaffolds by EDC involves the activation of a C-terminal, glutamate side chain and aspartate side chain carboxyl groups, and the formation of amide bonds upon coupling with amine groups of lysine. EDC/NHS leads to the formation of intermolecular and intramolecular cross-links (26). Grover et al. (31) have shown that cross-linking of gelatin-based scaffolds can improve mechanical strength and modulate degradation resistance providing scaffolds with increased structural integrity.

After crosslinking, we immobilized the peptide from amine groups using excess carboxyl groups that activated in the preceding step. It has been shown that gelatin has approximately 12.3% excess carboxyl (D and E) groups than amine (K) groups (32). Therefore, when gelatin is crosslinked to the degree that the ninhydrin test becomes negative, the majority of amine groups are coupled with carboxyl groups. Excess carboxyl groups can be used for peptide immobilization and further functionalization. To immobilize the target peptide or other biomolecules, different strategies have been used. In this regard, in the synthesized peptide we introduced two lysine (K) residues in order to couple the peptide using lysine side chain amines to carboxyl groups activated in the preceding cross-linking step in gelatin molecules. Gabriel et al. (33) have used a three-step procedure to immobilize RGD peptide on polycaprolactone film. This procedure involves amination, reaction with hetero-bifunctional cross-linkers and conjugation of an RGD-motif-containing peptide to the film. In the present study we have used a simple one step cross linking and immobilization procedure. With this procedure we employed an approach that not only cross-linked the scaffold but also provided activated carboxyl groups for immobilization of the peptide in the next step without any additional modification.

Although this peptide has the same composition as gelatin, a modified iodinated phenylalanine must be used to detect the immobilized peptide on the scaffold. FTIR studies confirmed these findings (Fig 4).

The results confirmed that immobilization of the peptides on the scaffold significantly enhanced hBMSCs adhesion and proliferation. Immobilization of cell adhesion motifs has been known to improve the surface chemistry of biomaterials. Recently, a large number of RGD-containing peptides with different sequences from ECM proteins have been synthesized (25). These peptides are immobilized on several polymers by a series of physical and chemical procedures. Many of these peptides have the RGD peptide sequence of fibronectin with some variations. Santiago et al. (34) have modified polycaprolactone disks with a three laminin- derived peptide sequence using carbodiimide chemistry. These investigators used aminolysis of polycaprolactone disks to introduce amine groups on the disks. We synthesized an RGD peptide from the origin of collagen IV. For the first time, in the current study, a peptide modification applied to gelatin-based nanofibrous scaffold was used. It has been shown that immobilization of RGDcontaining peptides on nanofibrous biomaterials improved cell adhesion and cell-matrix interaction (35, 36). Zhang et al. (37) studied the interaction between hBMSCs and a RGD-modified porous scaffold. These researchers observed that immobilization of RGD on a PCL scaffold improved hBMSC attachment and cellular distribution. They also found that integrin-mediated signal transduction pathways were significantly up-regulated by RGD modification. Activation of these pathways resulted in cell survival and growth.

The results of the cell adhesion assay showed that the PCL/Gel scaffold modified with designed peptide had better adhesion potential than PCL/ Gel itself. This modified scaffold could be used on biomaterial surfaces that have poor cell adhesion potential. Immobilization of the peptide has a dramatic effect on cell proliferation, which is important in fabricating biomaterials useful for regenerative medicine.

## Conclusion

A collagen IV-derived RGD-containing peptide was successfully synthesized and directly immobilized on a PCL/Gel scaffold pre-activated in the preceding cross-linking step. An iodine tag was used to track immobilization of the peptide. In order to confirm the adhesion properties of the synthesized peptide, we performed a cell adhesion assay. The results confirmed that the immobilization of the peptide on PCL/Gel scaffolds was successfully accomplished and could increase the cell adhesion and proliferation. Given the results of this study, engraftments of PCL/Gel by this novel peptide could increase the biocompatibility and suitability of PCL/Gel scaffolds for stem cells cultures.
